# An Explorative Study of the Incidental High Renal Excretion of [^18^F]PSMA-1007 for Prostate Cancer PET/CT Imaging

**DOI:** 10.3390/cancers14092076

**Published:** 2022-04-21

**Authors:** Youssra Allach, Amina Banda, Willemijn van Gemert, Michel de Groot, Yvonne Derks, Melline Schilham, Alexander Hoepping, Lars Perk, Martin Gotthardt, Marcel Janssen, James Nagarajah, Bastiaan M. Privé

**Affiliations:** 1 Department of Radiology and Nuclear Medicine, Radboud University Medical Centre, 6525 GA Nijmegen, The Netherlands; y.allach@erasmusmc.nl (Y.A.); amina.banda@radboudumc.nl (A.B.); willemijn.vangemert@radboudumc.nl (W.v.G.); michel.degroot@radboudumc.nl (M.d.G.); yvonne.derks@radboudumc.nl (Y.D.); melline.schilham@radboudumc.nl (M.S.); lars.perk@radboudumc.nl (L.P.); martin.gotthardt@radboudumc.nl (M.G.); marcel.janssen@radboudumc.nl (M.J.); james.nagarajah@radboudumc.nl (J.N.); 2 Department of Cardiology, Erasmus MC, 3015 CE Rotterdam, The Netherlands; 3 Department of Medicinal Chemistry, ABX Advanced Biochemical Compounds GmbH, 1454 Radeberg, Germany; hoepping@abx.de

**Keywords:** prostate cancer, urological oncology, [^18^F]PSMA-1007, PET, urinary uptake

## Abstract

**Simple Summary:**

Prostate cancer is one of the most dominant cancers in the Western world. In recent years, the use of positron emission tomography (PET) targeting prostate-specific membrane antigen (PSMA) to image prostate cancer allows accurate diagnosis and staging. Compared to the recently registered PET tracers [^68^Ga]Ga-PSMA-11 and [^18^F]DCFPyL, [^18^F]PSMA-1007 is predominantly excreted by the hepatobiliary tract, resulting in much lower urinary uptake. This allows for an improved evaluation of the pelvic area. However, on some occasions, clinicians do observe high excretion of [^18^F]PSMA-1007 by the renal system. Yet, this sudden differential metabolism of [^18^F]PSMA-1007 remains poorly understood. In this retrospective study, we aimed to elucidate the incidental high urinary uptake of [^18^F]PSMA-1007 by assessing the individual patient characteristics, scan (data) and peptide batches.

**Abstract:**

Positron emission tomography (PET) of prostate-specific membrane antigen (PSMA) allows for accurate diagnosis and staging of prostate cancer (PCa). Compared to other PSMA PET tracers available, [^18^F]PSMA-1007 is predominantly excreted via the hepatobiliary tract resulting in low renal excretion which improves evaluation of the pelvic area. However, some patients do show high urinary uptake of [^18^F]PSMA-1007. The present study aimed to investigate this sudden high urinary uptake of [^18^F]PSMA-1007 by evaluating [^18^F]PSMA-1007 PET scans from PCa patients. In this single-center retrospective study, patients that underwent [^18^F]PSMA-1007 PET imaging between July 2018 and January 2021 were included. Data regarding the individual patient characteristics, scan acquisition and batch production were analyzed. To determine the urinary excretion of [^18^F]PSMA-1007, a region of interest was drawn in the bladder, and standardized uptake values (SUVs) were calculated and compared to SUVs in the prostate. An SUVmax of >10 was considered high urinary excretion, an SUVmax 7.5–10 intermediate and an SUVmax < 7.5 low urinary excretion. A total of 344 patients underwent [^18^F]PSMA-1007 PET/CT imaging, with 37 patients receiving three or more [^18^F]PSMA-1007 PET/CT scans. The mean SUVmean and SUVmax of the bladder were 3.9 (SD 2.9) and 5.9 (SD 4.2), respectively. Fourteen percent of patients showed high urinary uptake of [^18^F]PSMA-1007. Twelve of the thirty-seven patients (32.4%) that had multiple scans showed a varying urinary uptake of [^18^F]PSMA-1007 per PSMA PET/CT scan. In terms of patient characteristics, risk factors, medication and blood laboratory results, no significant influencing variables were found. Nor was there a difference observed in the batch size and the mean radiochemical purity of PSMA-1007 for high- and low-excreting patients. However, the bladder volume affected the mean SUVmax in the bladder significantly, with higher SUVs in lower bladder volumes. In this study, we observed that a higher SUV in the urinary tract seemed to occur in patients with low bladder volume. A prospective study is needed to corroborate this hypothesis.

## 1. Introduction

In the last few decades, prostate cancer became one of the most dominant cancers, with more than a million new cases every year [1,2]. At present, the incidence of prostate cancer differs worldwide but is higher in developed countries, partly due to the larger availability of prostate-specific antigen (PSA) screening. With the Westernization and increasing life expectancy of the global population, it is anticipated that prostate cancer will remain an important health issue [3,4,5].

In recent years, the use of positron emission tomography (PET) probes targeting prostate-specific membrane antigen (PSMA) to image prostate cancer (metastases) has attracted broad attention [6,7,8]. Numerous studies have shown that commonly used PET radiotracers such as [^68^Ga]Ga-PSMA-11, [^18^F]DCFPyL and [^18^F]PSMA-1007 provide promising opportunities to localize prostate cancer and its metastases [2,9,10,11,12]. The use of this imaging modality improves the management of prostate cancer patients as it outperforms conventional imaging (e.g., computer tomography, bone scintigraphy) [9,11,13,14,15].

An important limitation of [^68^Ga]Ga-PSMA-11 and [^18^F]DCFPyL imaging is their high renal excretion, causing substantial tracer accumulation in the urinary tract such as the bladder, which hinders the detection of lesions in proximity (e.g., the prostate). This is particularly inconvenient with the current trend of using PSMA PET imaging to evaluate the local prostate tumor [10,16,17]. Compared to [^68^Ga]Ga-PSMA-11 and [^18^F]DCFPyL, [^18^F]PSMA-1007 is mainly excreted via the hepatobiliary tract resulting in much lower urinary uptake and thus allows for improved evaluation of the pelvic area where most prostate cancer lesions are located [10,16,17]. Interestingly, an increased excretion of [^18^F]PSMA-1007 by the renal system is observed in some patients [10]. At present, this sudden change in pharmacokinetics of [^18^F]PSMA-1007 remains poorly understood. We therefore aimed to elucidate this unexpected higher urinary uptake of [^18^F]PSMA-1007 by assessing individual patient characteristics, scan (data) and peptide batches.

## 2. Methods

### 2.1. Study Design and Participants

In this single-center retrospective study, all [^18^F]PSMA-1007 PET/CT scans acquired at Radboud University Medical Centre, Nijmegen, The Netherlands, between July 2018 and January 2021 were evaluated. The study was approved by the Medical Review Ethics Committee Arnhem-Nijmegen, The Netherlands (CMO 2020-7244), and all study subjects provided written informed consent and were registered in the ImPRINT registry (Radboudumc, CMO 2017-3275). All patient characteristics and scan data were collected and recorded in a validated electronic case report form (https://www.castoredc.com/, accessed on 5 February 2021).

### 2.2. Individual Patient

Characteristics, Pre-Treatment PSMA PET Data and Peptide Batches

The following individual patient characteristics were recorded: age of onset, all medication (e.g., diuretics), blood (tumor) markers (e.g., creatinine, liver function with alanine aminotransferase (ALAT) and aspartate aminotransferase (ASAT), PSA), disease stage and histopathology data. Risk factors were also collected such as: hypertension, diabetes, kidney disease and liver disease. Detailed definitions of the collected individual patient characteristics are summarized in Table S1.

The following [^18^F]PSMA-1007 PET/CT scan data were recorded: date and time of scan, the administered activity [^18^F]PSMA-1007, use of iodinated contrast, standardized uptake values (SUVs) of healthy organs (kidney parenchyma and pelvis, bladder, liver, spleen, salivary glands and healthy bone), bladder volume and increased general bone uptake. Moreover, the number, location and SUV of tumors were reported.

The following tracer data were recorded: batch production, batch size, cold mass of batch PSMA-1007 and radioactivity due to [^18^F]PSMA-1007 (HPLC).

### 2.3. Image Analysis

PET/CT images were reconstructed by Agfa Healthcare Impax (v6.6.1.5003) integrated with Oasis software (Segami Corporation, Columbia, MD, USA). All scans were evaluated following PSMA-RADS by board-certified nuclear medicine physicians with >10 years of experience. To measure the physiological uptake of [^18^F]PSMA-1007 in the kidney parenchyma and pelvis, bladder (center), liver, spleen, salivary glands and healthy bone (hip or acromion), a 20 mm region of interest (ROI) was positioned in each of these organs. In smaller regions, the ROI was reduced to 10 mm (e.g., kidney pelvis). The bladder volume was calculated by the following formula: length × height × width × π/6 (0.523) as commonly used in practice and described previously [18]. All the observed (tumor) lesions were reported and categorized into local recurrence, lymph node metastases, bone metastases and visceral metastases. Moreover, the scans were grouped based on the number of observed tumor lesions in <5, 5–20 and >20. For quantitative examination, a 20 mm region of interest (ROI) was placed on these lesions for the SUVmax and SUVmean. In case of multiple lesions per organ (local/lymph node/bone/visceral), the one with the highest SUVmax and SUVmean was recorded.

To assess whether a higher bone metabolism of [^18^F]PSMA-1007 was linked to high urinary uptake, the bone uptake of each scan was categorized into three groups by examining the maximum intensity projection (MIP): tracer uptake below the blood pool, tracer uptake equivalent to the blood pool and tracer uptake higher than the blood pool (Figure S1). Patients with very extensive bone metastases were excluded from this analysis.

### 2.4. Statistical Analyses

Data were managed according to Good Clinical Practice requirements using a validated data capture system (https://www.castoredc.com/, accessed on 5 February 2021). Descriptive statistical methods were used to characterize the study cohort. Stratified data were compared using the chi-squared or Fisher’s exact test for categorical variables and the Mann–Whitney U and Kruskal–Wallis test for continuous variables.

Pearson’s chi-squared or Fisher’s exact test was performed to compare groups of categorical data. All analyses were performed using IBM SPSS Statistics, version 25.0 (IBM Corp., Armonk, NY, USA). In all analyses, a *p*-value below 0.05 was considered significant, and only two-sided *p*-values were used for the reported analyses. The applied statistical analyses are listed in the figure and table legend. Figures were generated using GraphPad Prism (version 5.03; GraphPad Software Inc., La Jolla, CA, USA).

## 3. Results

### 3.1. Baseline Demographics

A total of 344 of the studied patients (*n* = 344 men; 100%) underwent [^18^F]PSMA-1007 PET/CT imaging between July 2018 and January 2021 and met the inclusion criteria. Eleven patients were excluded due to a lack of data in the medical records (including their PSMA PET/CT). Of the total study population, 78, 26, 8 and 3 patients had two, three, four and five PSMA PET scans, respectively. The mean age at the time of the PSMA PET scan was 71 years (SD 7.2 years). Of all included patients, 228 (66.1%) received prior radiotherapy, 225 (65.2%) androgen deprivation therapy, 155 (44.9%) radical prostatectomy and 53 (15.4%) chemotherapy. Hypertension was the most common risk factor (35.7%), followed by diabetes (8.7%) and kidney disease (7.2%; Table S2). Between the injection of [^18^F]PSMA-1007 and the scan, it took an average of 83 min (SD 21 min). The [^18^F]PSMA-1007 (mean) administered activity was 253 MBq (SD 16.1). Iodinated contrast medium was administered to 350 patients (69.3%). None of the patients received loop diuretics (e.g., furosemide) prior to the PET scan. The mean SUVmean and SUVmax of the bladder were 3.9 (SD 2.9) and 5.9 (SD 4.2), respectively. The SUVmean and SUVmax of local prostate cancer lesions were 8.1 (SD 7.5) and 24.8 (SD 23.1), respectively.

A total of 259 (98.1%) scans had a local prostate cancer lesion that was easily delineable with a higher SUVmax of the tumor than the SUVmean of the bladder. Sixty-six scans (13.7%) in 35 patients had an SUVmax > 10 in the bladder urine. The clinical characteristics of scans of patients with high (SUVmax > 10), intermediate (SUVmax 7.5–10) and low urinary (SUVmax < 7.5) excretion of [^18^F]PSMA-1007 are presented in Table 1.

### 3.2. Patient Characteristics

There were no significant influencing factors identified in disease-related parameters, risk factors, medication, and blood laboratory results (Table 1). Patients with high urinary excretion of [18F]PSMA did not use more diuretics compared to low excreting individuals (16.9% vs. 10.9%, respectively; *p* = 0.167). This was also the case for ACE inhibitors (4.6% vs. 11.5%, respectively; *p* = 0.097). Furthermore, there was no association between high urinary uptake of [18F]PSMA-1007 and the existence of kidney disorders. However, >20 tumor lesions appeared to be more prevalent in individuals with high urinary uptake of [18F]PSMA-1007 compared to those with low urinary uptake (27.3 vs. 14.4%, respectively, *p* = 0.017).

**Figure 1:**
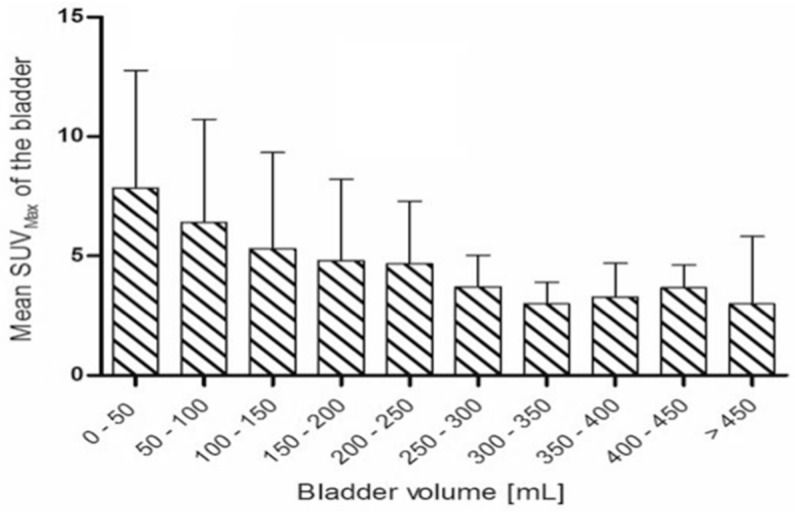
Mean SUVmax (SD) of the bladder stratified by bladder volume. SUV: standardized uptake value; SD: standard deviation.

The median bladder volume was 67 mL [IQR 46–97] and significantly smaller in the patient group with an SUVmax of >10.0 (*p* ≤ 0.001) (Table 1). Hence, the mean SUVmax in the bladder decreased in a higher-volume bladder, as seen in Figure 1.

**Figure 2:**
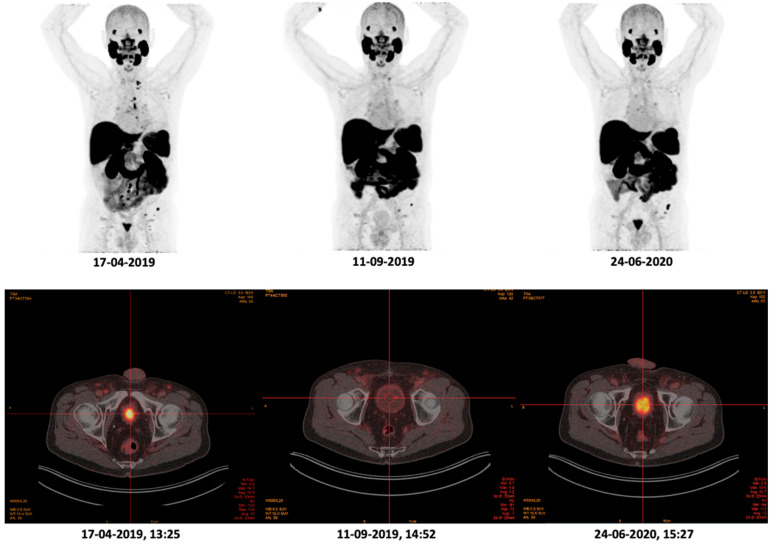
Maximum intensity projections and fused images of the urinary bladder uptake on [18F]PSMA-1007 PET/CT. This patient had an SUVmax (of the bladder) and bladder volume of 15.7 and 24 mL, respectively, in the first scan (17 April 2019), an SUVmax of 2.0 and bladder volume of 109 mL in the second scan (11 September 2019) and an SUVmax of 13.6 and bladder volume of 54 mL in the third scan (24 June 2020). Between these PET/CT scans, there were no changes in the patients’ medication use, blood laboratory results or introduction of new risk factors. PSMA: prostate-specific membrane antigen; PET: positron emission tomography; CT: computed tomography; MIP: maximum intensity projection; SUV: standardized uptake value; PSA: prostate-specific antigen.

Of the total study population, 37 patients received three or more [^18^F]PSMA-1007 PET/CT scans. Twelve of these patients (32.4%) showed a differing level of urinary uptake with low and high excretion of [^18^F]PSMA-1007 between scans. Figure 2 depicts such a patient with varying urinary uptake of [^18^F]PSMA-1007 per PSMA PET/CT scan. Between these PET/CT scans, there were no changes in the patients’ medication use, blood laboratory results or introduction of new risk factors. Furthermore, no correlations were discovered based on (pre-)scan data (e.g., administered activity and batch size of [^18^F]PSMA-1007). However, studying the individual scans, it is noticeable that a smaller bladder volume was observed in the case of high urinary uptake compared to the scans without a high uptake, as can be seen in Figure 2. For each of the 37 patients’ individual patient and scan data are presented in Table S4.

### 3.3. Scan Characteristics

**Figure 3:**
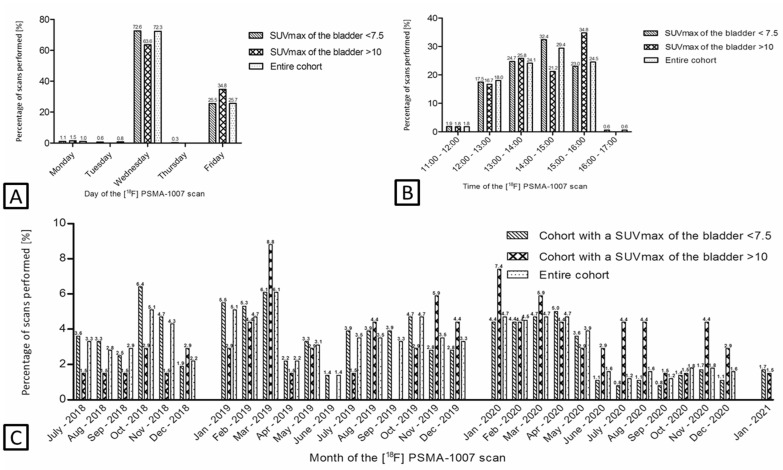
Percentage of scans performed for the cohort with an SUVmax of the bladder < 7.5, SUVmax of the bladder >10 and for the entire cohort stratified by day (**A**), time (**B**) and month (**C**) of the [^18^F]PSMA-1007 PET/CT. PSMA: prostate-specific membrane antigen; SUV: standardized uptake value.

**Figure 4:**
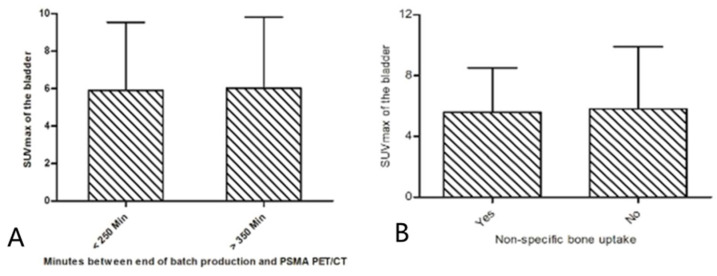
Mean SUVmax (SD) of the bladder stratified by minutes between batch production and PSMA PET/CT (**A**) and presence of increased general bone uptake (**B**). SD: standard deviation; PSMA: prostate-specific membrane antigen; PET: positron emission tomography; CT: computed tomography; SUV: standardized uptake value; PSA: prostate-specific antigen; Min: minute.

The administered activity [^18^F]PSMA-1007 was a mean of 257 MBq (SD 19.8) in the high-excreting and 253 MBq (SD 15.4) in the low-excreting scans (*p* = 0.162). There were no significant variations observed in the administration of contrast media between the high-excreting and low excreting patients (60.6% vs. 70.0%, respectively; *p* = 0.130). Figure 3 shows the percentage of scans of the cohort with an SUVmax of the bladder <7.5, the high [^18^F]PSMA renal excreting scans (SUVmax of >10.0) and the entire cohort classified by time of the scan, the day of the week and the months since the start of [^18^F]PSMA PET scans. This was to evaluate if certain dates resulted in higher urinary uptake (e.g., certain batch productions). In general, no relation was observed regarding the date or time of the PET/CT scan; most of the high-excreting scans were performed on a Wednesday (63.6%), yet this observation did not differ when looking at the entire cohort as most scans were performed on Wednesday (72.3%). However, when comparing the time of the scan with the entire cohort, we observed that high-excreting scans were more frequently performed at the end of the day (3:00–4:00 p.m.) and thus with a longer time between batch production and injection (34.8% vs. 24.5%, respectively). In Table S3, the median SUVmax of the bladder [IQR] is stratified per month. Forty-three scans (9.8%) showed an increased general bone uptake of [^18^F]PSMA-1007 (Table S5). No statistically significant differences were observed when the SUVmax in the bladder was stratified for the presence of increased general bone uptake (*p* = 0.099) (Figure 4B).

### 3.4. [^18^F]PSMA-1007 Radio-Labeling Characteristics

All batches were produced around 9 am (SD 28 min). The average batch size was 48.1 GBq (SD 12.3) and 46.5 GBq (SD 13.4) for high- and low-excreting patients, respectively (*p* = 0.297). There was no difference in time between injection of [^18^F]PSMA-1007 and PET/CT of high-excreting and low-excreting patients (82 min (SD 20 min) vs. 83 min (SD 21 min), respectively; *p = 0.242*). The time between batch production and PSMA PET/CT was 301 min (SD 74 min) vs. 297 min (SD 67 min) for the high- and low-excreting scans, respectively. Figure 4A shows the mean SUVmax in the bladder stratified for <250 min and >350 min between batch production and [^18^F]PSMA-1007 PET/CT scan. No difference was observed in the mean radiochemical purity of PSMA-1007 for high- and low-excreting patients (98.4% (SD 1.2%) vs. 98.3% (SD 1.3%)). Table S2 provides more data on [^18^F]PSMA-1007 batch production.

## 4. Discussion

[^18^F]PSMA-1007 is a novel PSMA radiotracer for imaging of prostate cancer patients. Currently, the registration trial of [^18^F]PSMA-1007 is recruiting patients globally (NCT0S). [^18^F]PSMA-1007 has a particular advantage over the other PSMA tracers due to a low urinary uptake, which improves evaluation of tumors in proximity of the urinary tract. This is particularly wanted with the current movement to evaluate the local prostate tumor with PSMA PET imaging [10,16,17]. However, we observed that 14% of patients do show high urinary uptake of [^18^F]PSMA-1007. In an attempt to define which characteristics contribute to this presently undetermined increased urinary uptake, this study evaluated individual patient characteristics, (pre-)scan (data) and production batch data of [^18^F]PSMA-1007 PET scans.

According to our findings, an incremental urinary uptake was not due to medication, certain diseases (e.g., kidney or liver), altered scan protocols, administered activity of [^18^F]PSMA-1007 or due to batch productions. However, a higher [^18^F]PSMA-1007 uptake was seen in patients with less urine volume in the bladder. We assume this is caused by a higher concentration of [^18^F]PSMA-1007 in urine, resulting in higher SUV values on PET imaging. Based on this finding, we suggest adequate fluid intake and no voiding prior to the scan to dilute the [^18^F]PSMA-1007 in the urine with non-active already present urine. A reasonably filled bladder also has a better contour, which improves the delineation of surrounding organs such as the prostate and seminal vesicles. This hypothesis conflicts with the guideline published by the Society of Nuclear Medicine and Molecular Imaging (SNMMI) and the European Association of Nuclear Medicine (EANM) which recommends voiding directly prior to [^68^Ga]Ga-PSMA-11 and [^18^F]DCFPyL PET acquisition to reduce the activity present in the urinary system [6]. However, this recommendation is based on tracers eliminated via the urinary system. Furthermore, individuals who had a high urinary uptake of [^18^F]PSMA-1007 had more (tumor) lesions (>20) than individuals with low urinary uptake. This relationship is unclear to us, since a possible tumor sink effect with unfavorable tracer distribution to the healthy organs in a low-volume disease setting would protect from high renal wash-out [19]. The significance of our observation may therefore be a coincidental finding.

While it was a hypothesis prior to the study, we cannot associate (spontaneous) proteinuria to a higher urinary uptake of [^18^F]PSMA-1007 as neither kidney diseases nor increased creatinine levels were found to affect the SUV of the bladder [20]. The present study also evaluated if a certain time of the PET scan was related to higher urinary uptake of the tracer (e.g., specific batch productions). Our data show that no such relationship exists, other than that it seems that high-excreting scans were more frequently performed between 3:00 p.m. and 4:00 p.m. However, this was not related to a longer time between batch production and scan acquisition time, as these did not differ between low- and high-excreting patients.

Thus, this observation seems to be unrelated to potential radiolysis resulting in free ^18^F. Several studies have also reported that no radiolysis occurs with [^18^F]PSMA-1007 in the first eight hours after the synthesis [21]. Moreover, defluorination of ^18^F labelled PSMA, or free ^18^F, causes non-specific bone absorption and should influence the evaluation of the skeleton [22,23]. Yet, there was no evidence for a link to increased general bone uptake. However, correction for the physical decay of ^18^F requires application of higher peptide dosages, which may have resulted in more peptide presentation to the kidneys. A variation in the peptide concentration or molar activity of the injected tracer could affect wash-out of the radiotracer [24]. This will need evaluation in a prospective setting to adequately correct for this. All in all, only a marginal increase in high-urinary-excreting scans was observed at the end of the day, which could still be a coincidence.

Furthermore, while PSMA is highly overexpressed in most prostate cancers and is clinically visualized using PSMA-specific probes, a recent study by Bakht et al. showed that the correlation is not perfect and there may also be active tracer uptake in tissue with low PSMA expression [25]. Therefore, the incidental uptake may also be due to non-specific uptake of [^18^F]PSMA-1007 to still unknown targets.

The study has certain limitations: First, the study is constrained by its retrospective collection of data. Second, since our cohort represents a heterogeneous group, we cannot exclude a variety of potential biases that may have affected the outcomes. Third, only limited data of the cold mass were available, as collection of this parameter was difficult. In addition, it would have been appealing to have urine samples post injection to measure the amount of [^18^F]PSMA-1007 and control for proteinuria. Unfortunately, this needs to be done in a prospective trial. An additional study is therefore needed to really elucidate the differential urinary uptake between [^18^F]PSMA PET scans and to test the present outcomes in a more controlled setting.

## 5. Conclusions

In conclusion, we observed that patient characteristics or scanning procedures did not result in a high urinary uptake of [^18^F]PSMA PET in the bladder. However, higher SUVs in the urinary tract occurred in patients with low bladder volume. Therefore, adequate fluid intake and no voiding prior to the scan may improve the detection of tumors in proximity of the bladder. Enhanced tracer uptake in the bladder occurs in just a small subset of individuals, and it is therefore unclear how widely this clinical advice should be used. A prospective study is now needed to evaluate the present observations in more detail and to confirm this new hypothesis.

## Figures and Tables

**Table 1:** Demographic patient characteristics stratified by SUVmax of the bladder.

Patient Characteristics	SUVmax ≤ 7.5	SUVmax 7.5–10.0	SUVmax > 10	*p* *
Number of scans, *n* (%)	361 (74.7)	56 (11.6)	66 (13.7)	
Mean age, years ± SD	70.7 ± 7.2	72.1 ± 7.4	71.3 ± 7.4	*0.654* *
Median PSA at diagnosis, ng/mL (IQR)	14 (8.1–34.9)	12 (8.7–20.3)	12.6 (6.8–59.8)	*0.991* *
Median PSA before scan, ng/mL (IQR)	7.8 (1.6–24.5)	3.5 (1.2–14.0)	4.3 (0.7–29.8)	*0.022* *
Iodinated contrast media, *n* (%)	250 (70.0)	43 (76.8)	40 (60.6)	*0.130* *
Mean number of minutes between acquisition time [^18^F]PSMA-1007 and PSMA PET/CT scan ± SD	83 ± 21	80 ± 17	82 ± 20	*0.594* *
Mean amount of administered activity [^18^F]PSMA-1007, MBq ± SD	253 ± 15.4	249 ± 13.0	257 ± 19.8	*0.162* *
Bladder				
Mean SUVmean ± SD	2.7 ± 1.2	5.5 ± 1.3	8.9 ± 4.0	≤*0.001* *
Mean SUVmax ± SD	3.9 ± 1.6	8.7 ± 0.7	14.2 ± 4.3	≤*0.001* *
Median bladder volume, ml (IQR)	106 (68–181)	64 (45–89)	67 (46–97)	≤*0.001* *
Left Kidney				
Mean SUVmean parenchyma ± SD	13.4 ± 3.6	13.7 ± 3.3	13.9 ± 3.3	*0.339* *
Mean SUVmax parenchyma ± SD	27.3 ± 6.6	28.1 ± 6.9	27.3 ± 8.2	*0.555* *
Mean SUVmean pelvis ± SD	2.6 ± 0.8	2.5 ± 0.7	2.8 ± 0.9	*0.429* *
Mean SUVmax pelvis ± SD	3.5 ± 1.1	3.4 ± 1.0	3.8 ± 1.2	*0.326* *
Right Kidney				
Mean SUVmean parenchyma ± SD	14.3 ± 7.4	14.1 ± 3.0	14.7 ± 4.3	*0.575* *
Mean SUVmax parenchyma ± SD	27.2 ± 6.8	28.1 ± 6.0	27.3 ± 8.4	*0.392* *
Mean SUVmean pelvis ± SD	2.9 ± 0.7	2.9 ± 0.7	3.1 ± 0.8	*0.446* *
Mean SUVmax pelvis ± SD	3.8 ± 1.0	3.7 ± 0.9	4.2 ± 1.3	*0.020* *
Liver				
Mean SUVmean ± SD	11.5 ± 3.1	11.2 ± 2.8	11.1 ± 3.9	*0.526* *
Mean SUVmax ± SD	14.4 ± 3.8	14.1 ± 3.3	14.1 ± 4.7	*0.520* *
Spleen				
Mean SUVmean ± SD	11.0 ± 4.1	11.7 ± 4.4	10.7 ± 3.9	*0.375* *
Mean SUVmax ± SD	14.0 ± 4.9	14.8 ± 5.3	13.5 ± 4.7	*0.540* *
Salivary glands				
Mean SUVmean ± SD	15.4 ± 5.3	16.5 ± 5.2	15.2 ± 5.4	*0.712* *
Mean SUVmax ± SD	30.1 ± 9.3	30.8 ± 9.8	29.8 ± 9.4	*0.976* *
Healthy bone				
Mean SUVmean ± SD	0.3 ± 1.1	0.2 ± 0.07	0.2 ± 0.08	*0.628* *
Mean SUVmax ± SD	0.6 ± 2.2	0.4 ± 0.1	0.5 ± 0.2	*0.616* *
Biopsy histology, *n* (%)				
ISUP Grade 1 (Gleason score 3 + 3 = 6)	28 (11.6)	4 (11.4)	2 (6.3)	*0.384* *
ISUP Grade 2 (Gleason score 3 + 4 = 7)	41 (17.0)	8 (22.9)	10 (31.3)	*0.042* *
ISUP Grade 3 (Gleason score 4 + 3 = 7)	52 (21.6)	7 (20.0)	6 (18.8)	*0.768* *
ISUP Grade 4 (Gleason score = 8)	61 (25.3)	10 (28.6)	6 (18.8)	*0.462* *
ISUP Grade 5 (Gleason score = 9–10)	58 (24.1)	6 (17.1)	7 (21.9)	*0.846* *
General bone uptake, *n* (%)				
Above the blood pool	31 (9.7)	7 (14.3)	4 (7.4)	*0.586* *
Equivalent to the blood pool	148 (46.5)	27 (55.1)	27 (50)	*0.638* *
Below the blood pool	139 (43.7)	15 (30.6)	23 (42.6)	*0.878* *
Suspicious lesions/tumors, *n* (%)	328 (91.1)	49 (87.5)	55 (83.3)	*0.054* *
Number of suspicious lesions/tumors, *n* (%)				
<5	171 (52.5)	28 (57.1)	24 (43.6)	*0.226* *
5–20	108 (33.1)	15 (30.6)	16 (29.1)	*0.554* *
>20	47 (14.4)	6 (12.2)	15 (27.3)	*0.017* *
Location of suspicious lesions/tumors, *n* (%)				
Local recurrence	200 (55.4)	26 (46.4)	31 (47.0)	*0.206* *
Bone	159 (44.0)	19 (33.9)	29 (43.9)	*0.987* *
Lymph node	177 (49.0)	33 (58.9)	37 (56.1)	*0.294* *
Visceral	19 (5.3)	3 (5.4)	3 (4.5)	*0.808* *
Type of therapy, *n* (%)				
Radical prostatectomy	111 (43.9)	15 (41.7)	16 (45.7)	*0.837* *
Radiotherapy	163 (64.4)	24 (66.7)	26 (74.3)	*0.250* *
Cryoablation	11 (4.3)	3 (8.3)	1 (2.9)	*0.679* *
ADT (with or without chemotherapy)	165 (65.2)	22 (61.1)	24 (68.6)	*0.695* *
Chemotherapy	40 (15.8)	2 (5.6)	8 (22.9)	*0.294* *
Radium-223	13 (5.1)	1 (2.8)	4 (11.4)	*0.139* *
PSMA radioligand therapy	22 (8.7)	2 (5.6)	3 (8.6)	*0.980* *
Risk factors, *n* (%)				
Hypertension	94 (37.2)	11 (30.6)	10 (28.6)	*0.322* *
Diabetes	21 (8.3)	3 (8.3)	3 (8.6)	*0.957* *
Kidney disease	21 (8.3)	0 (0)	1 (2.9)	*0.256* *
Hepatic disease	1 (0.4)	0 (0)	0 (0)	*0.709* *
Baseline blood parameters				
Mean hemoglobin, mmol/L ± SD	8.0 ± 1.1	8.6 ± 0.8	8.2 ± 1.0	*0.565* *
Mean creatinine, umol/L ± SD	91.8 ± 33.4	95.0 ± 51.1	79.6 ± 17.8	*0.315* *
Mean glomerular filtration rate, mL/min/1.73 m^2^ ± SD	74.3 ± 15.3	74.0 ± 18.8	81.2 ± 11.9	*0.063* *
Median lactate dehydrogenase, U/I (IQR)	205(185.5–245.5)	209.5 (178.8–257)	192 (134–217.8)	*0.505* *
Median alkaline phosphatase, U/I (IQR)	82 (64–114)	84 (64–148)	83 (57–110)	*0.829* *
Medication, *n* (%)				
Antihypertensive drugs	134 (38.4)	19 (34.5)	20 (30.8)	*0.243* *
ACE inhibitors	40 (11.5)	10 (18.2)	3 (4.6)	*0.097* *
Diuretics	38 (10.9)	7 (12.7)	11 (16.9)	*0.167* *
Prednisone	25 (7.2)	1 (1.8)	5 (7.7)	*0.880* *
Calcium carbonate	57 (16.3)	5 (9.1)	5 (7.7)	*0.073* *
Biphosphonates	7 (2.0)	1 (1.8)	2 (3.1)	*0.587* *
Chemotherapy	2 (0.6)	0 (0)	1 (1.5)	*0.399* *
[^18^F]PSMA-1007 batch				
Mean number of minutes between batch production and acquisition time of [^18^F]PSMA-1007 ± SD	214 ± 65	218 ± 61	219 ± 70	*0.242* *
Mean number of minutes between batch production and acquisition time of [^18^F]PSMA-1007 PSMA PET/CT ± SD	297 ± 67	297 ± 62	301 ± 74	*0.691* *
Mean batch size, GBq ± SD	46.5 ± 13.4	49.9 ± 12.3	48.1 ± 12.3	*0.297* *
Mean cold mass of batch, mg/mL ± SD	0.004 ± 0.004	0.005 ± 0.004	0.004 ± 0.004	*0.802* *
Mean radiochemical purity ± SD	9833 ± 1.3	98.4 ± 1.1	98.4 ± 1.2	*0.432* *

Data are numbers (%), mean (SD), median [IQR] or *p*-values. Percentages are the proportion of patients with that specific factor based on the total patients with available data of the certain factor. SD: standard deviation; IQR: interquartile range; PSA: prostate-specific antigen; PSMA: prostate-specific membrane antigen; MBq: megabecquerel; PET: positron emission tomography; CT: computed tomography; ISUP: International Society of Urological Pathologists; ADT: androgen deprivation therapy; GBq: gigabecquerel. * Statistical analyses were performed based on the group SUV max ≤ 7.5 and the group SUV max > 10.

## Data Availability

All patient characteristics and scan data were collected and recorded in a validated electronic case report form (https://www.castoredc.com/, accessed on 5 February 2021) and are available upon reasonable request to the corresponding author.

## Supplementary Materials

The following supporting information can be downloaded at: https://www.mdpi.com/article/10.3390/cancers14092076/s1, Figure S1: Maximum intensity projections of the [18F]PSMA-1007 tracer uptake in three patients; Table S1: Definitions of the risk factors, Table S2: Baseline characteristics of the total study population, Table S3: Median SUVmax of the bladder stratified by date (month) of the scan, Table S4: individual patient characteristics and scan characteristics of patients that underwent thee or more [18F]PSMA-1007 PET/CT scans, Table S5: Demographic patient characteristics stratified by increased general bone uptake.
