# Supplementary material for: An Explorative Study of the Incidental High Renal Excretion of [18F]PSMA-1007 for Prostate Cancer PET/CT Imaging
Source: Cancers (Basel). 2022 Apr 21;14(9):2076. doi: 10.3390/cancers14092076 (PMC9100267; doi:10.3390/cancers14092076)
Supplement: Supplementary file 1 [file cancers-14-02076-s001.zip › cancers-1664787-supplementary.pdf]

Supplementary Materials: An Explorative Study of the Incidental High Renal Excretion of [^{18}F]PSMA-1007 for Prostate Cancer PET/CT Imaging

Youssra Allach, Amina Banda, Willemijn van Gemert, Michel de Groot, Yvonne Derks, Melline Schilham, Alexander Hoepping, Lars Perk, Martin Gotthardt, Marcel Janssen, James Nagarajah and Bastiaan M. Privé

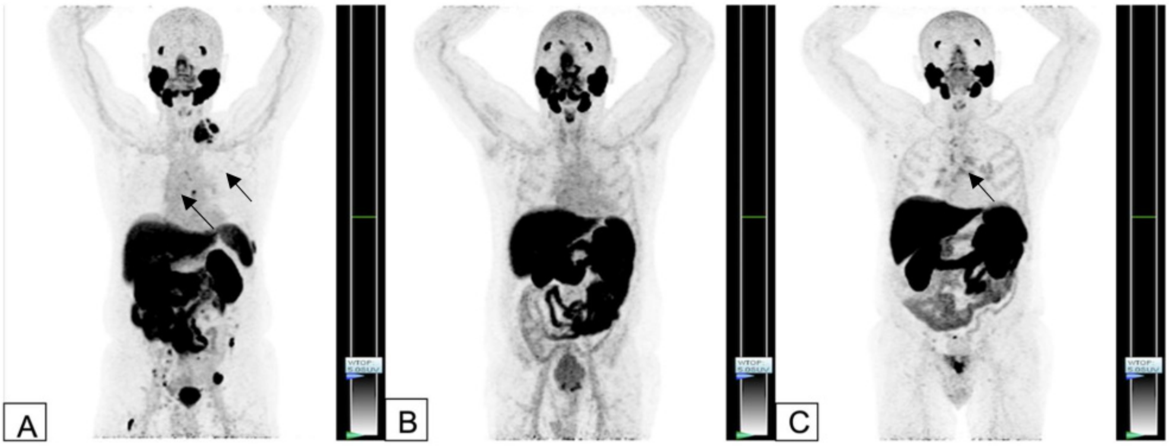

Figure S1. Maximum Intensity Projections of the [^{18}F]PSMA-1007 tracer uptake in three patients. (**A**)Tracer uptake below the blood pool, (**B**)Tracer uptake equivalent to the blood pool, and (**C**) Tracer uptake higher than the blood pool. The arrows indicate the area that we referred as higher/lower uptake than the blood pool.

Table S1. Definitions of the risk factors.

Variable or Risk Factor	Definition
Hypertension	A history of hypertension was defined as the presence of hypertension in the patient's medical history, the use of antihypertensive medication, and/or systolic and diastolic blood pressures of 140 mm Hg and 90 mm Hg, respectively.
Diabetes mellitus	Diabetes was defined as the existence of the disease in the patient's medical history or when diabetic medication was used.
Kidney disease	The presence of kidney disease in a patient's medical history was classified as a history of renal disease.
Hepatic disease	The presence of hepatic illness in a patient's medical history was classified as a history of hepatic disease.
ADT (with or without chemotherapy)	Abiraterone tablet<br>Leuprorelin injection powder wwsp<br>Gosereline implant wwsp<br>Buserline implant wwsp<br>Bicalutamide tablet<br>Enzalutamide tablet
Antihypertensive drugs	Metoprolol succinate tablet<br>Metoprolol tartrate tablet<br>Candesartan tablet<br>Valsartan tablet<br>Losartan tablet<br>Irbesartan tablet<br>Telmisartan tablet<br>Hydralazine tablet<br>Nifedipine tablet<br>Propranolol tablet<br>Atenolol tablet<br>Chlortalidone tablet<br>Bisoprolol tablet

	Amlodipine tablet<br>Lercanidipine tablet<br>Barnidipine tablet<br>Doxazosin tablet
ACE inhibitors	Quinapril tablet<br>Lisinopril tablet<br>Enalapril tablet<br>Perindopril tablet<br>Fosinopril tablet
Diuretics	Hydrochlorothiazide tablet<br>Eplerenone tablet<br>Furosemide tablet<br>Indapamide tablet<br>Bumetanide tablet<br>Spironolactone tablet<br>Amiloride/hydrochlorothiazide tablet
Prednisone	Prednisone tablet<br>Prednisolone tablet
Calcium carbonate	Calcium/cholecalciferol chew tablet<br>Calciumcarbonate/colecalcif granulate<br>Denosumab injvlst<br>Calcium chew tablet (calci chew)<br>Natriumbicarbonate<br>Carbasalaatcalcium powder<br>Calciumacetaat/magnesiumcarbonate
Biphosphates	Zoledronic acid<br>Alendronic acid tablet<br>Risedronic acid tablet
Chemotherapy	Cabazitaxel<br>Docetaxel

Table S2. Baseline characteristics of the total study population.

**Characteristics**	
Number of patients, *n (%)*	344 (100%)
Number of scans, *n (%)*	505 (100%)
Mean age of patients, years ± SD	71 ± 7.2
Median PSA at diagnosis, ng/ml (IQR)	14.0 (7.9 – 34.2)
Median PSA before scan, ng/ml (IQR)	7.2 (1.5 – 22.3)
Iodinated contrast media, *n (%)*	350 (69.3)
Mean number of minutes between acquisition time [^{18}F]PSMA-1007 and PSMA PET/CT scan ± SD	83 ± 21
Mean amount of administered activity [^{18}F]PSMA-1007, MBq ± SD	253 ± 16.1
Bladder	
Mean SUV_{mean} ± SD	3.9 ± 2.9
Mean SUV_{max} ± SD	5.9 ± 4.2
Median Bladder volume, *ml (IQR)*	90 (59.8 – 158.3)
Left Kidney	
Mean SUV_{mean} parenchyma ± SD	13.5 ± 3.6
Mean SUV_{max} parenchyma ± SD	27.3 ± 6.9
Mean SUV_{mean} pelvis ± SD	2.6 ± 0.8
Mean SUV_{max} pelvis ± SD	3.6 ± 1.1
Right Kidney	
Mean SUV_{mean} parenchyma ± SD	14.3 ± 6.6

Mean SUV_{max} parenchyma ± SD	27.2 ± 7.0
Mean SUV_{mean} pelvis ± SD	2.9 ± 0.7
Mean SUV_{max} pelvis ± SD	3.9 ± 1.
Liver	
Mean SUV_{mean} ± SD	11.4 ± 3.2
Mean SUV_{max} ± SD	14.3 ± 3.9
Spleen	
Mean SUV_{mean} ± SD	11.4 ± 7.3
Mean SUV_{max} ± SD	14.1 ± 5.0
Salivary gland	
Mean SUV_{mean} ± SD	15.5 ± 5.3
Mean SUV_{max} ± SD	30.2 ± 9.3
Healthy bone	
Mean SUV_{mean} ± SD	0.3 ± 1.0
Mean SUV_{max} ± SD	0.5 ± 1.9
Biopsy histology, n (%)	
ISUP Grade 1 (Gleason score 3 + 3 = 6)	36 (11.1)
ISUP Grade 2 (Gleason score 3 + 4 = 7)	61 (18.8)
ISUP Grade 3 (Gleason score 4 + 3 = 7)	67 (20.7)
ISUP Grade 4 (Gleason score = 8)	81 (25)
ISUP Grade 5 (Gleason score = 9 - 10)	79 (24.4)
General bone uptake, *n (%)*	
Above the blood pool	43 (9.8)
Equivalent to the blood pool	212 (48.5)
Below the blood pool	182 (41.6)
Suspicious lesions/tumours, *n (%)*	448 (90.0)
Number of suspicious lesions/tumours, *n (%)*	
<5	227 (50.9)
5-20	148 (33.2)
>20	71 (15.9)
Location of suspicious lesions/tumours, *n (%)*	
Local recurrence	264 (51.7)
Bone	213 (41.7)
Lymph node	260 (50.9)
Visceral	26 (5.1)
Type of therapy, *n (%)*	
Radical prostatectomy	155 (44.9)
Radiotherapy	228 (66.1)
Cryoablation	20 (5.8)
ADT (with or without chemotherapy)	225 (65.2)
Chemotherapy	53 (15.4)
Radium-223	18 (5.2)

PSMA radioligand therapy	30 (8.7)
Risk factors, *n (%)*	
Hypertension	123 (35.7)
Diabetes	30 (8.7)
Kidney disease	25 (7.2)
Hepatic disease	1 (0.3)
Baseline Blood parameters	
Mean Haemoglobin, *mmol/l ± SD*	8.1 ± 1.0
Mean Creatinine, *umol/l ± SD*	90.7 ± 33.9
Mean Glomerular filtration rate, *ml/min/1,73m2 ± SD*	74.9 ± 15.4
Median Lactate dehydrogenase, *U/l (IQR)*	201 (184 – 246)
Median Alkaline phosphatase, *U/l (IQR)*	82 (65 – 110)
Medication, *n (%)*	
Antihypertensive drugs	183 (37.2)
ACE inhibitors	53 (10.8)
Diuretics	59 (12.0)
Prednisone	33 (6.7)
Calcium carbonate	68 (13.8)
Bisphosphonates	10 (2.0)
Chemotherapy	3 (0.6)
[^{18}F]PSMA-1007 batch	
Mean batch size, *GBq ± SD*	46.9 ± 13.1
Mean cold mass of batch, *mg/mL ± SD*	0.004 ± 0.004
Mean Radiochemical purity ± *SD*	98.3 ± 1.3

Data are numbers (%), mean (SD) or median [IQR]. Percentages are the proportion of patients with that specific factor based on the total patients with available data of the certain factor. SD: Standard deviation; IQR: Interquartile range; PSA: Prostate-specific Antigen; MBq: Mega Becquerel; ISUP: International Society of Urological Pathologists; ADT: androgen deprivation therapy; GBq: Giga-becquerel. PSMA: Prostate specific membrane antigen; PET: Positron emission tomography; CT: Computed tomography; SUV: Standardized uptake value.

Table S3. Median SUV_{max} of the bladder stratified by date (month) of the scan.

Month of scan	Number of scans, *n (%)*	Number of scans with high urinary uptake (SUVmax > 10), *n (%)*	Median SUV $_{max}$ of bladder [IQR]
July – 2018	17 (3.3)	1 (1.5)	3.5 [2.4 – 5.2]
Aug – 2018	14 (2.8)	1 (1.5)	2.8 [2.3 – 4.8]
Sep – 2018	15 (2.9)	1 (1.5)	3.5 [2.4 – 5.5]
Oct – 2018	26 (5.1)	2 (2.9)	4.0 [2.3 – 6.2]
Nov – 2018	22 (4.3	1 (1.5)	2.9 [2.3 – 4.7]
Dec – 2018	11 (2.2)	2 (2.9)	4.4 [3.5 – 8.3]
Jan – 2019	26 (5.1)	2 (2.9)	5.2 [2.6 – 7.4]
Feb – 2019	24 (4.7)	3 (4.4)	4.3 [2.8 – 6.0]
Mar – 2019	31 (6.1)	6 (8.8)	4.0 [3.4 – 7.5]
Apr – 2019	11 (2.2)	1 (1.5)	4.6 [3.0 – 7.6]
May – 2019	16 (3.1)	2 (2.9)	3.7 [2.5 – 7.8]
June – 2019	7 (1.4)	0 (0)	5.4 [3.6 – 7.3]
July – 2019	18 (3.5)	1 (1.5)	4.0 [2.9 – 6.4]
Aug – 2019	18 (3.5)	3 (4.4)	4.1 [3.4 – 6.7]
Sep – 2019	17 (3.3)	0 (0)	2.7 [2.0 – 3.8]
Oct -2019	24 (4.7)	2 (2.9)	3.9 [3.1 – 8.0]
Nov – 2019	18 (3.5)	4 (5.9)	6.8 [4.7 – 9.3]

Dec – 2019	17 (3.3)	3 (4.4)	6.4 [3.5 – 9.4]
Jan – 2020	24 (4.7)	5 (7.4)	5.8 [4.1 – 9.1]
Feb – 2020	23 (4.5)	3 (4.4)	4.2 [2.3 – 9.1]
Mar – 2020	24 (4.7)	4 (5.9)	3.9 [2.9 – 9.1]
Apr – 2020	24 (4.7)	3 (4.4)	4.4 [3.0 – 7.2]
May – 2020	20 (3.9)	2 (2.9)	5.7 [3.4 – 9.3]
June – 2020	8 (1.6)	2 (2.9)	4.9 [2.0 – 10.0]
July – 2020	6 (1.2)	3 (4.4)	10.2 [3.8 – 10.2]
Aug – 2020	8 (1.6)	3 (4.4)	6.7 [3.9 – 13.0]
Sep – 2020	6 (1.2)	1 (1.5)	5.3 [3.4 – 20.2]
Oct – 2020	9 (1.8)	1 (1.5)	7.0 [5.4 – 9.4]
Nov – 2020	9 (1.8)	3 (4.4)	6.0 [4.0 – 11.8]
Dec – 2020	8 (1.6)	2 (2.9)	7.7 [5.9 – 10.7]
Jan – 2021	8 (1.6)	1 (1.5)	5.7 [4.6 – 7.6]

Data are numbers (%) or median [IQR]. Percentages are the proportion of scans with that factor based on the total scans with available data of the certain factor. SUV: standardized uptake value; IQR: Interquartile range.

Table S4. Individual patient and scan characteristics of patients that underwent three or more [^{18}F]PSMA-1007 PET/CT scans.

Patients that underwent Three, Four or Five [^{18}F]PSMA-1007 PET/CT Scans	SUV_{mean} bladder	SUV_{max} bladder	Bladder volume (mL)	Creatinine (umol/l)	Glomerular filtration rate (GFR)	Antihypertensive drugs	Diuretics	Non-specific bone uptake	Minutes between scan and acquisition of [^{18}F]PSMA - 1007	Minutes between scan and batch production	Administered activity (MBq)	Batch size (GBq)	HPLC
11001													
1st scan	1.2	1.9	149	58	90	No	No	No	104	398	274	35.66	95.1
2nd scan	2.3	3.5	161	68	90	Yes	No	No	70	202	261	41.85	98
3rd scan	2.4	3.5	234	76	86	Yes	No	No	86	249	232	45.02	-
11002													
1st scan	2.4	3.2	95			Yes	No	Yes	76	239	249	31.02	-
2nd scan	6.2	10.5	96	103		Yes	No	Yes	63	205	246	66.60	100
3rd scan	2.1	3.3	72			Yes	No	Yes	113	400	249	47.37	97
11003													
1st scan	1.3	2.4	52			Yes	No	-	98	383	265	45.94	99
2nd scan	4.1	6.6	64			Yes	No	-	111	259	251	57.33	98
3rd scan	1.5	2.0	90			Yes	No	No	75	334	241	44.60	99
11004													
1st scan	4.5	5.8	64	95	68	Yes	Yes	No	67	211	260	41.02	98.3
2nd scan	6.4	9.0	71	80	83	Yes	Yes	No	52	207	246	66.10	100
3rd scan	7.5	15.2	49	72	87	Yes	Yes	No	68	345	245	35.02	-
11005													
1st scan	1.3	2.2	124			No	No	No	119	229	236	13.96	96.2
2nd scan	1.3	1.8	65			No	No	No	94	269	268	31.02	100
3rd scan	1.7	2.3	102	75	90	No	No	No	66	301	258	51.60	99
11006													
1st scan	5.2	15.7	24			No	No	No	120	265	285	37.85	98
2nd scan	1.3	2.0	109	96	68	No	No	No	106	349	249	63.50	99
3rd scan	8.6	13.6	54	97	67	No	No	No	98	383	248	66.40	98
11007													
1st scan	3.9	6.7	186			Yes	Yes	Yes	57	180	241	36.46	97.2
2nd scan	3.7	5.5	237	99	68	Yes	Yes	Yes	109	346	242	66.60	100
3rd scan	5.2	8.5	225			Yes	Yes	Yes	98	244	236	61.50	-
11008													

1st scan	6.6	8.9	51			No	No	No	73	312	268	34.5	99.1
2nd scan	4.5	5.9	60			No	No	No	71	394	245	61.00	99
3rd scan	5.1	8.9	61			No	No	No	81	217	251	31.91	-
11009													
1st scan	2.4	3.5	64	111	58	No	No	No	118	270	258	41.57	96.3
2nd scan	4.6	7.3	42			No	No	No	71	326	285	44.42	100
3rd scan	2.3	3.6	50	98	66	No	No	No	109	231	250	61.50	-
11010													
1st scan	3.1	5.3	165	87	74	No	No	Yes	86	359	235	63.32	98
2nd scan	2.6	4.8	135	99	64	No	No	Yes	81	275	249	44.40	-
3rd scan	7.1	10.5	134	98	64	No	No	Yes	93	404	258	48.60	98
11011													
1st scan	2.9	4.0	88	62	90	Yes	No	No	64	270	245	31.6	97.8
2nd scan	2.4	3.7	156	62	90	Yes	No	No	124	225	254	25.70	97.5
3rd scan	4.6	14.0	35	51	90	Yes	No	No	58	220	254	41.85	98
11012													
1st scan	3.0	4.0	93	76	88	Yes	Yes	No	147	265	265	48.02	99
2nd scan	10.5	12.4	64	67	90	Yes	Yes	-	100	376	275	68.49	100
3rd scan	3.1	8.4	73			Yes	Yes	-	94	383	237	45.00	98
11013													
1st scan	1.4	2.3	114	108		No	No	Yes	122	260	250	21.61	98.2
2nd scan	4.7	7.1	86	87	80	No	No	Yes	102	231	264	43.10	99
3rd scan	5.0	11.2	21	90	76	No	No	Yes	94	348	220	31.91	-
11014													
1st scan	9.5	14.1	96	85		Yes	No	No	143	396	260	36.46	97.2
2nd scan	8.2	11.4	99	88	79	Yes	No	No	94	229	254	30.50	100
3rd scan	2.5	7.9	131			Yes	No	No	72	214	260	48.60	98
11015													
1st scan	3.3	4.6	154	83	78	Yes	No	Yes	84	270	251	52.11	97
2nd scan	1.7	2.7	139			Yes	No	Yes	90	197	242	67.45	100
3rd scan	2.0	3.0	58	88	73	Yes	No	Yes	79	252	249	66.40	99
11016													
1st scan	0.9	1.4	64			No	No	No	119	350	262	35.02	96.7
2nd scan	1.7	2.6	74			No	No	No	75	336	261	53.28	99
3rd scan	1.9	2.6	52			No	No	No	81	388	263	47.37	97
11017													
1st scan	1.7	2.9	277	78		No	No	No	97	391	266	29.4	99

2nd scan	3.9	5.3	142	80	84	No	No	No	55	303	259	39.70	-
3rd scan	3.4	4.3	208	87	77	No	No	No	53	285	249	52.20	95.9
11018													
1st scan				81	81	No	No	No			244	31.98	95.9
2nd scan	2.5	4.5	81	78	85	No	No	No	102	314	260	36.46	97.2
3rd scan	6.2	11.4	83	73	90	No	No	No	107	372	247	32.76	99.2
11019													
1st scan	8.6	13.1	82	68	90	No	No	Yes	96	226	245	52.33	98
2nd scan	5.3	7.5	83			No	No	Yes	97	282	247	52.06	99
3rd scan	7.0	9.4	126	82	85	No	No	Yes	82	246	259		-
11020													
1st scan	7.0	11.8	111			No	No	No	82	260	269	62.22	100
2nd scan	12.0	14.7	162			No	No	No	77	375	232	41.70	98
3rd scan	5.5	13.1	89					No	67	328	238	52.04	95
11021													
1st scan	2.6	3.7	380			No	No	No	68	403	257	49.6	99
2nd scan	4.8	6.6	231	79	83	No	No	No	109	243	254	68.49	100
3rd scan	2.4	3.6	157			No	No	No	97	217	237	41.70	98.5
11022													
1st scan	11.8	14.7	92			Yes	Yes	No	98	403	250	32.89	98.1
2nd scan	3.0	4.1	86			Yes	Yes	No	79	304	243	35.02	96.7
3rd scan	12.0	14.8	186			Yes	Yes	No	52	152	237	44.40	97.5
11023													
1st scan	2.3	3.2	184			No	No	No	119	311	287	32.89	98.1
2nd scan	4.1	5.3	118			No	No	No	71	301	232	42.17	98
3rd scan	4.9	6.9	49			No	No	No	77	202	236	62.30	98.4
11024													
1st scan	1.4	2.3	98			No	No	No	96	223	277	31.21	96.2
2nd scan	2.4	7.9	63	63	90	No	No	No	64	283	265	48.39	99
3rd scan	1.6	2.3	106	69	90	No	No	No		259	270	45.85	96
11025													
1st scan	3.7	5.5	78	81	86	No	No	-	111	325	246	37.34	96.5
2nd scan	4.7	8.1	64			No	No	-	67	192	255	55.24	99
3rd scan	23.6	32.2	61	79	88	No	No	-	75	331	220	36.00	97.3
11026													
1st scan	2.2	3.1	91			Yes	No	No	94	335		64.66	100
2nd scan	5.2	8.8	751			Yes	No	No	52	364	244	51.60	99

3rd scan	4.3	6.0	125			Yes	No	No	86	368	245	45.04	95.6
11027													
1st scan	3.6	5.5	70			No	No	No	102	370	261	31.21	96.2
2nd scan	2.7	4.5	70			No	No	No	105	226	260	23.20	99.1
3rd scan	6.2	8.6	106			No	No	No	79	376	245	52.33	98
4th scan	2.4	3.8	62			No	No	No	57	195	233	48.37	100
11028													
1st scan	1.1	1.7	1356	76	90	No	No	No	137	290	247	48.02	99
2nd scan	2.4	4.0	114			No	No	No	87	310	241	52.06	99
3rd scan	1.6	2.3	128			No	No	No	98	284	251	39.17	99
4th scan	4.5	6.4	132			No	No	No	81	223	224	45.04	95.6
11029													
1st scan	3.7	5.1	50	69	90	No	No	No	116	414	273	13.69	96.2
2nd scan	3.2	5.3	31	76	90	No	No	No	79	195	255	46.90	100
3rd scan	3.3	8.8	28			No	No	No	72	234	272	55.50	99
4th scan	2.2	3.1	158			No	No	No	97	351	250	45.00	98.8
11030													
1st scan						No	No	-	94	215	241	42.5	100
2nd scan						No	No	Yes	82	208	250	57.50	100
3rd scan						No	No	No	56	205	231	66.40	98
4th scan						No	No	No	79	215	264	45.50	97
11031													
1st scan	4.9	8.1	37			No	No	No	98	269	261	18.44	95.7
2nd scan	7.3	10.3	52			No	No	No	83	200	269	48.90	99
3rd scan	14.1	17.3	58			No	No	No	81	233	225	55.50	97.9
4th scan	18.5	24.2	50			No	No	No	90	265	260	45.50	97
11032													
1st scan	9.8	11.8	81			No	No	No	74	220	235	70	99
2nd scan	12.5	17.8	42			No	No	No	90	129	282	57.33	98
3rd scan	9.4	17.7	39			No	No	No	86	254	225	35.50	98
4th scan	3.6	4.6	162			No	No	No	89	390	233	36.00	97.3
11033													
1st scan	0.9	1.4	273			Yes	No	-	77	384	268	38.31	94
2nd scan	2.0	3.1	283	120	51	Yes	No	No	79	248	252	52.11	97
3rd scan	2.5	3.4	302			Yes	No	No	79	297	243	47.78	99
4th scan	0.9	1.4	376	147	40	Yes	No	No	56	328	256	47.40	99
11034													

1st scan	1.6	3.0	86	107	61	No	No	No	75	317	254	44.42	100
2nd scan	2.3	3.8	67	93	71	No	No	No	112	251	263	42.17	98
3rd scan	1.1	2.0	186	102	54	No	No	No	53	210	246	67.40	97
4th scan	4.8	6.4	84	94	70	No	No	No	96	388	245	52.43	97
11035													
1st scan	4.2	6.3	51	80	83	No	No	No	104	268	258	32.89	98.1
2nd scan	4.0	5.4	57	74	90	No	No	No	89	243	260	42.61	99.5
3rd scan	4.5	6.6	68			No	No	No	90	264	253	68.49	100
4th scan	4.9	8.1	55			No	No	No	92	211	241	52.36	99
5th scan	6.6	10.4	76	85	77	No	No	Yes	74	419	279	48.0	96.1
11036													
1st scan	4.8	7.2	36			No	No	No	72	230	270	29.2	99
2nd scan	4.7	8.4	17			No	No	No	85	222	260	68.49	100
3rd scan	5.9	10.2	35	82	81	No	No	No	94	418	249	63.32	98
4th scan	5.1	10.0	30	90	72	No	No	No	77	283	249	45.00	98
5th scan	3.2	5.3	33			No	No	No	94	258	249	48.6	98
11037													
1st scan	2.2	3.2	198			No	No	No	123	308	252	21.61	98.2
2nd scan	3.1	4.1	154			No	No	No	101	369	245	25.70	97.5
3rd scan	3.9	6.4	54			No	No	No	89	293	258	49.89	99
4th scan	1.6	2.2	87			No	No	No	70	197	247	35.50	98
5th scan	2.6	3.8	106			No	No	No	92	253	232	53.2	97.9

Individual patient, scan, and batch characteristics. MBq: Mega Becquerel; GBq: Giga-becquerel; PSMA: Prostate specific membrane antigen; PET: Positron emission tomography; CT: Computed tomography; SUV: Standardized uptake value; HPLC: radioactivity due to $[^{18}F]$PSMA-1007.

Table S5. Demographic patient characteristics stratified by increased general bone uptake.

**Increased General Bone Uptake**	**Yes**	**No**	***p***
Number of cases, *n (%)*	43 (9.8)	394 (90.2)	
Mean age, years ± SD	70.0 ± 7.3	70.1 ± 7.1	*0.929*
Median PSA at diagnosis, ng/ml (IQR)	13 (7.8 – 27.8)	13 (7.6 – 31.4)	*0.372*
Iodinated contrast media, *n (%)*	33 (78.6)	278 (70.9)	*0.296*
Mean amount of administered activity ^{18}F-PSMA-1007, MBq ± SD	252 ± 13.1	252 ± 15.7	*0.486*
Bladder			
Mean SUV $_{mean}$ ± SD	3.6 ± 1.9	3.8 ± 2.7	*0.133*
Mean SUV $_{max}$ ± SD	5.6 ± 2.9	5.8 ± 4.1	*0.099*
Median Bladder volume, ml (IQR)	105 (72 – 170)	92 (60 – 157)	*0.547*
Liver			
Mean SUV $_{mean}$ ± SD	13.4 ± 3.9	11.2 ± 2.9	*0.103*
Mean SUV $_{max}$ ± SD	16.5 ± 4.5	14.2 ± 3.6	*0.318*
Healthy bone			
Mean SUV $_{mean}$ ± SD	0.2 ± 0.06	0.3 ± 1.1	*0.587*
Mean SUV $_{max}$ ± SD	0.4 ± 0.1	0.6 ± 2.1	*0.577*
Suspicious lesions/tumours, *n (%)*	37 (88.1)	347 (88.5)	*0.935*
Number of suspicious lesions/tumours, *n (%)*			
<5	20 (55.6)	202 (58.4)	*0.744*
5–20	15 (41.7)	119 (34.4)	*0.384*
>20	1 (2.8)	25 (7.2)	*0.313*
Risk factors, *n (%)*			
Hypertension	12 (48.0)	97 (36.5)	*0.255*
Diabetes	3 (12.0)	23 (8.6)	*0.574*
Kidney disease	0 (0)	17 (6.4)	*0.193*
Hepatic disease	0 (0)	1 (0.4)	*0.759*
Medication, *n (%)*			
Antihypertensive drugs	20 (47.6)	138 (36.1)	*0.144*
ACE inhibitors	5 (11.9)	41 (10.7)	*0.817*
Diuretics	4 (9.5)	42 (11.0)	*0.771*
Prednisone	0 (0)	22 (5.8)	*0.110*
Calcium carbonate	1 (2.4)	43 (11.3)	*0.073*
Bisphosphonates	3 (7.1)	2 (0.5)	*<0.001*
Chemotherapy	2 (4.8)	1 (0.3)	*<0.001*
[^{18}F]PSMA-1007 batch			
Mean number of minutes between batch production and acquisition time of [18F]PSMA-1007 ± SD	189 ± 61	218 ± 65	0.332
Mean number of minutes between batch production and [^{18}F]PSMA-1007 PSMA PET/CT ± SD	276 ± 58	300 ± 68	0.089
Mean Batch size, GBq ± SD	47.1 ± 14.2	46.9 ± 13.2	0.584
Mean cold mass of batch, mg/mL ± SD	0.006 ± 0.004	0.004 ± 0.004	0.315
Mean Radiochemical purity ± SD	98.2 ± 1.3	98.3 ± 1.3	0.555

Data are numbers (%), mean (SD), median [IQR] or p-values. Percentages are the proportion of patients with that specific factor based on the total patients with available data of the certain factor. SD: Standard deviation; IQR: Interquartile range; PSA: Prostate-specific Antigen; PSMA: Prostate specific membrane antigen; MBq: Mega Becquerel; PET: Positron emission tomography; CT: Computed tomography; GBq: Giga-becquerel.
